# PREDICTORS OF MEDICAL, SOCIAL SERVICE, AND MENTAL HEALTH USE AMONG PEOPLE WITH DEMENTIA AND THEIR CAREGIVERS

**DOI:** 10.1093/geroni/igz038.3396

**Published:** 2019-11-08

**Authors:** Srijana Shrestha, Amber Amspoker, Tracy L Evans, Melinda Stanely, Jessica Freshour, Sheila Richey, Mark E Kunik

**Affiliations:** 1 Wheaton College, MA, Norton, Massachusetts, United States; 2 Baylor College of Medicine, Houston, Texas, United States; 3 Michael E. DeBakey VA Medical Center, Houston, Texas, United States

## Abstract

People with Alzheimer’s disease use more medical services (Eaker et al., 2002) and are admitted to inpatient facilities at higher rates (Zhu et al., 2015) than normal controls. In addition, social services provide support for caregivers and are associated with positive outcomes for care-recipients and their caregivers (Neville, Beattle, Fielding, & MacAndrew, 2014). Despite high level of need, utilization of mental health services and social agencies for caregiver support remains low (Goodarzi, Mele, Roberts & Holroyd-Leduc, 2017; Weber, Pirraglia & Kunik, 2011). Following the Andersen and Newman model (1973), we examined whether predisposing factors (i.e., age of the caregiver and type of PWD-caregiver relationship), needs (i.e., memory impairment, disruptive behaviors, depression, anxiety, pain, functional impairment, caregiver burden, total number of prescribed medications), and enabling factors (i.e., PWD and caregiver income, quality of the PWD-caregiver relationship) differentially predicted the presence of medical, social, and mental health service use. A total of 228 dyads (PWD and the caregiver) were included. We examined each PWD and caregiver characteristic individually (univariate models) and then as a unique predictor of each of the three service use outcomes (multivariate models). A greater number of medications uniquely predicted higher medical service use, greater pain severity and PWD income were uniquely associated with higher social service use, and a greater number of medications and increased memory impairment predicted more mental health service use. These results show that distinct factors predict use of different types of service use among PWD and their caregivers.

